# Distance-Dependent Fluorescence Resonance Energy Transfer Enhancement on Nanoporous Gold

**DOI:** 10.3390/nano11112927

**Published:** 2021-11-01

**Authors:** Lianmin Cui, Ling Zhang, Heping Zeng

**Affiliations:** 1 School of Optical-Electrical and Computer Engineering, University of Shanghai for Science and Technology, Shanghai 200093, China; lmcui@usst.edu.cn; 2 Public Experiment Center, University of Shanghai for Science and Technology, Shanghai 200093, China; 3 Key Laboratory for Ultrafine Materials of Ministry of Education, School of Materials Science and Engineering, East China University of Science and Technology, Shanghai 200237, China; hpzeng@phy.ecnu.edu.cn; 4 Chongqing Key Laboratory of Precision Optics, Chongqing Institute of East China Normal University, Chongqing 401120, China

**Keywords:** FRET, plasmon, nanoporous gold, fluorescence enhancement, protein

## Abstract

Fluorescence resonance energy transfers (FRET) between cyan fluorescent protein (CFP) and yellow fluorescent protein (YFP) on nanoporous gold (NPG) are systematically investigated by controlling the distance between NPG and fluorescent proteins with polyelectrolyte multilayers. The FRET between CFP and YFP is significantly enhanced by NPG, and the maximum enhancement is related to both ligament size of NPG and the distance between NPG and proteins. With the optimized distance, 18-fold FRET enhancement was obtained on NPG compared to that on glass, and the conversion efficiency is about 90%. The potential to tune the characteristic energy transfer distance has implications for applications in nanophotonic devices and provides a possible way to design sensors and light energy converters.

## 1. Introduction

Fluorescence resonance energy transfer (FRET) is a non-radiative energy transfer process based on dipole interaction [1,2,3], and it has attracted a great attention due to its application in detecting low concentrated analytes in chemical and biological systems [4,5,6,7,8]. However, the limitation of the signal amplification of acceptor from donor and relatively low energy conversion efficiency affect the application of FRET in ultra-sensitive detection [9]. Plasmonic enhancement which resulting from longer range non-radiative energy transfer via nanometal surface energy transfer or localized surface plasmon-coupled FRET effect is a promised method to further improve the fluorescence intensity of the acceptor, extending the application of FRET [10,11,12,13,14,15,16,17]. The FRET between different fluorophores can be modulated by adjacent plasmonic nanocrystals [18,19,20,21,22,23], and the efficiency of FRET and acceptor fluorescence intensity from FRET varies with the size, shape, and location of the metal nanostructure [24,25,26,27]. A two-fold improvement of energy transfer between donor and acceptor has been realized with silver film due to the strong confinement and large propagation length of surface plasmon polaritons [20]. The FRET efficiencies are significantly increased by more than 50% for Cy3–Cy5, and can be increased by up to 61% in G protein-coupled receptor probes in the gold nanoaperture [21,28]. A FRET signal was enhanced to be two times stronger for cell surface imaging and the acceptor QDs’ photoluminescence lifetime, increased from 3.38 to 7.52 ns with gold nanoparticles [25]. Nanoporous gold (NPG) with tunable three-dimensional porous structure has demonstrated significant fluorescence enhancement on organic fluorophores and semiconductor quantum dots (QDs) [29]. Here, we investigate the distance-dependent FRET enhancement on NPG using polyelectrolyte multilayers as dielectric spacers between fluorescent protein pairs and plasmonic nanostructures. With assembling of polyelectrolyte multilayers of poly(allylamine hydrochloride) (PAH) and poly(styrene sulfonate) (PSS) [30,31], distance-dependent FRET enhancement between donor CFP and acceptor YFP was studied with NPG as substrate. A maximum fluorescence emission was observed at a distance of ~10.5 nm from NPG, where the fluorescence intensity of the acceptor molecule (YFP) detected in the FRET process was improved 18-fold compared to that on glass, and the FRET efficiency was increased up to 90%.

## 2. Materials and Methods

### 2.1. Preparation of NPG Substrates

**Figure 1:**
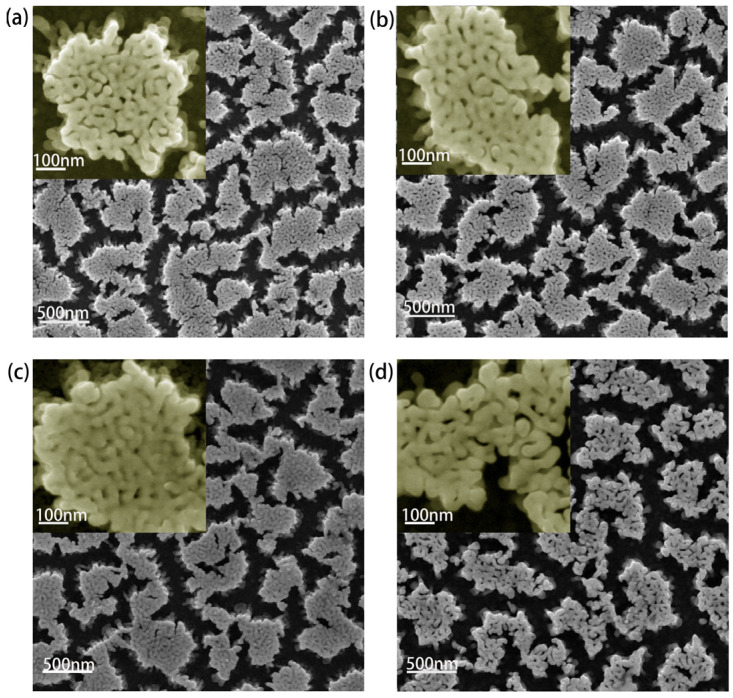
SEM micrographs of NPG with different Ligament sizes. The Ligament diameters are 27 nm (**a**), 32 nm (**b**), 38 nm (**c**), and 45 nm (**d**).

NPG substrates were prepared by dealloying an Au_25_Ag_75_ (wt.%) precursor sputtered on silicon wafer with a chromium layer and a gold layer for buffering. The magnetron sputtering (CFSP01) parameters were set as 1200 W power and 1Pa internal pressure to get uniform alloy films. With selectively etching silver component from the alloy by 68% nitric acid, an open bicontinuous three-dimensional nanoporous structure expands to the entire film, and the ligament and pore sizes increase with extending etching time [32,33,34,35]. NPG films with the average ligament sizes of 27 nm (NPG27), 32 nm (NPG32), 38 nm (NPG38) and 45 nm (NPG45) prepared with different etching time were used in the experiment, as shown in Figure 1. The composition analysis of the dealloyed samples was checked by an energy disperse spectroscopy (EDS, FEG250, Waltham, MA, USA). As shown in Table 1, after dealloying, the content of residual silver of the four samples is around 2%, where the influence on plamonic properties can be neglected, since residual silver in such amount always remains inside the gold ligament [36]. Additionally, with statistical analysis, the ratios of pore size to ligament diameter are about 0.45 (NPG27), 0.39 (NPG32), 0.34 (NPG38), and 0.64 (NPG45), respectively, and the one with small ratio is predicted to possess better plamonic enhancement [37].

### 2.2. Materials

Sodium salt of cysteamine was obtained from Aladdin (Shanghai, China). CFP and YFP were purchased from Biovision (Milpitas, CA, USA) incorporated, and phosphate buffer solution (PBS, pH value is 7.4) was obtained from Macklin (Shanghai, China). CFP and YFP freeze-dried powders were centrifuged and diluted in PBS. Deionized water, purified using a Millipore Milli-Q gradient system (UPR-11-10T, Chengdu, China) with a resistivity of 18.2 MΩ cm, was used for layer-by-layer (LbL) assembly. 

### 2.3. Polyelectrolyte Multilayers Layers Assembly 

**Figure 2:**
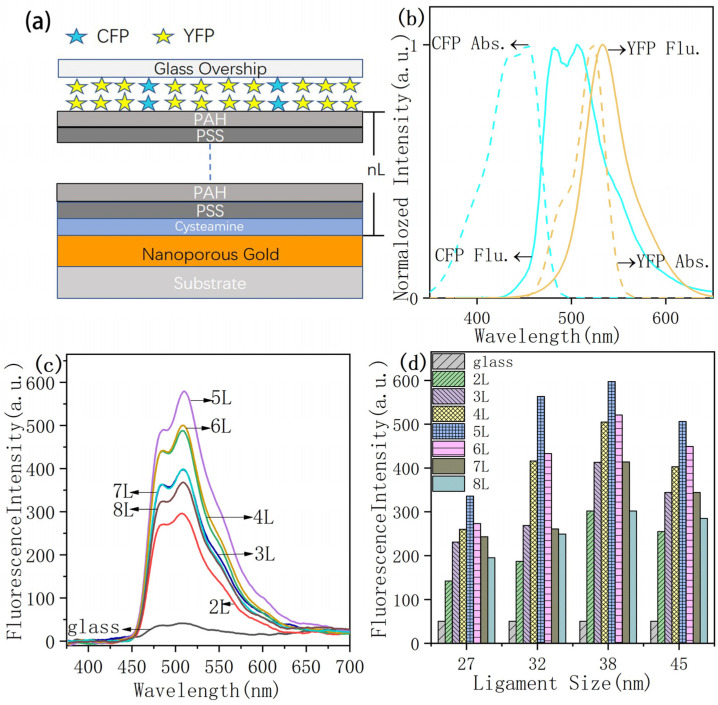
Scheme and spectrum. (**a**) Scheme of donor–acceptor assemble on the surface of glass and NPG. (**b**) Normalized absorption and fluorescence spectra of donor and acceptor for CFP and YFP. (**c**) Fluorescence spectra of donor (CFP) at 2–8 L to NPG films with NPG Ligament diameter of 38 nm. The black carve is the emission spectrum of CFP on glass slide. (**d**) The average peak value of CFP fluorescence intensity at 2–8 L to NPG films with NPG Ligament of 27 nm, 32 nm, 38 nm, and 45 nm.

In order to control the distance between proteins and NPG, polyelectrolyte multilayers were assembled on NPG surface, and 3 mg/mL PSS aqueous solution and 2 mg/mL PAH aqueous solution were used to form spacer layer. NPG films were incubated overnight with a 1 mM ethanolic solution of cysteamine to form a self-assembled monolayer of cysteamine on the surface of NPG films, resulting in an amine-NPG with a positive charge functionalized surface. Amine-NPG films and glass were used as substrates for further LbL assembly of PSS/PAH. Multilayer PSS and PAH (2–8 layers) were consecutively alternated adsorbed on amine-NPG surface [30,31,38,39], keeping PAH as the outermost layer, and then CFP and YFP were assembled outside. The thickness of the spacer layer was controlled by inserting different numbers of PSS/PAH layers. Since each layer of PSS/PAH assembly adds a distance of about 2.1 nm, 2 to 8 layers of PSS/PAH were assembled and formed approximately 4.2, 6.3, 8.4, 10.5, 12.6, 14.7 and 16.8 nm spacer distance between NPG and proteins. After PSS/PAH layers were completely dried at ambient conditions, CFP and YFP were diluted in phosphate buffer (pH 7.4), and a drop of 1µL solution with 3.2 × 10^−6^ M CFP and 3.2 × 5 × 10^−6^ M YFP was added onto the surface of each substrate (2 mm × 2 mm) to ensure the same amount of fluorophore proteins on the samples. Figure 2a shows the schematic preparation of protein adsorbed on LbL-assembled substrates.

### 2.4. Fluorescence Spectroscopy and Efficiency and Enhancement Factor of FRET

Microstructure characterization and material property analysis were accomplished by using a scanning electron microscope (SEM, FEG250, Waltham, MA, USA) and fluorescence spectrometer with 405 nm laser excitation. The laser power at the sample surface was about 200 µW and the exposure time was set at 1000 ms. Multiple fluorescence data were collected evenly on the same sample and averaged for analyzing. 

Efficiency of FRET (E) was calculated by using of Förster formula [40,41]:

E = 1 − F_DA_/F_D_ (1)

where F_D_ and F_DA_ were the donor’s fluorescence intensity measured in the absence and presence of acceptor, correspondingly.

For the convenience of expression, the FRET enhancement factor Q was defined by Formula (2),

Q = (F_AD_ (NPG) − F_A_ (NPG))/(F_AD_ (glass) − F_A_ (glass)) (2)

where F_A_ (NPG) and F_AD_ (NPG) were the measured fluorescence intensity of acceptor in the absence and presence of donor on NPG surface, and F_A_ (glass) and F_AD_ (glass) were measured florescence intensity in the absence and presence of donor on glass slide.

## 3. Results and Discussion

### 3.1. CFP Fluorescence (Donor)

Normalized absorption and emission spectra of CFP and YFP are shown in Figure 2b. Since NPG films have strong absorption range between 380 and 600 nm [42], it overlaps with the absorption ranges of CFP and YFP, which is benefit for plasmon resonance enhancement.

The distance-dependent fluorescence enhancement of CFP was firstly studied. The fluorescence emission spectra from CFP on NPG38 with varied probe distances tuned by PSS/PAH layers are shown in Figure 2c. The fluorescence intensity from CFP on NPG surface was significantly enhanced compared to that on a glass slide. The maximum enhancement value of 12-fold was exhibited at a distance of ~10.5 nm (5L) from NPG38 (Figure 2d). 

### 3.2. FRET Based on NPG

**Figure 3:**
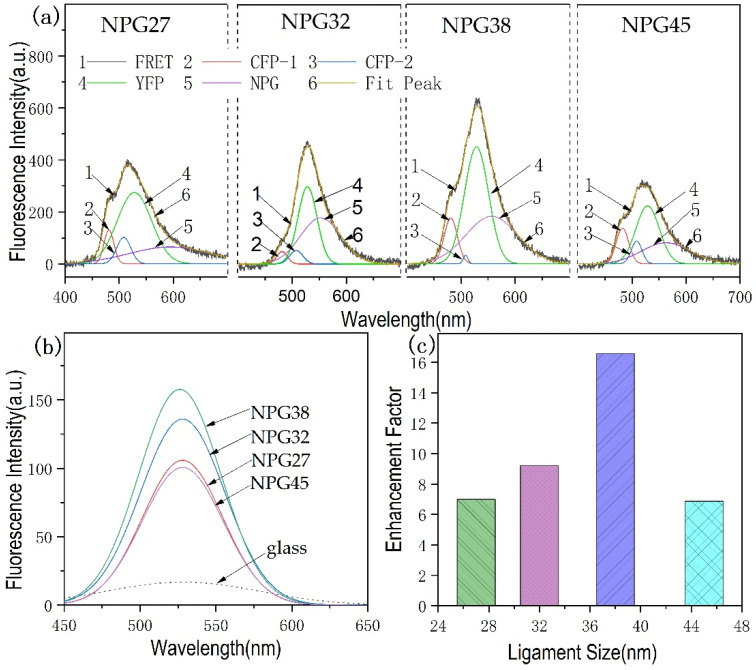
Fluorescence spectra and Enhancement Factor. (**a**) Fluorescence spectra of donor (CFP)-acceptor (YFP) on NPG with different ligament sizes when the spacing distance is around 10.5 nm. (**b**) Fluorescence spectra of YFP only on NPG with different ligament sizes and glass slide. (**c**) Enhancement factor of YFP with CFP and NPG substrate.

Since a 10.5 nm spacer layer shows the best fluorescence enhancement of CFP, the FRET was measured on NPG films with 5L PSS/PAH layers for spacing. Figure 3a shows the florescence spectra of YFP-CFP on NPG27, NPG32, NPG 38 and NPG45 films. In order to facilitate the analysis, we divided the FRET emission spectrum into four spectra CFP-1(480), CFP-2(508), YFP(528) and NPG resonance peaks by peak fitting in Origin. Comparing the four pictures, it can be seen that the acceptor molecular (YFP) had the strongest fluorescence emission, while the fluorescence intensity of the donor molecule (CFP) dropped to the smallest when the ligament size of NPG is 38 nm.

For comparison, the fluorescence of YFP only on 5L PSS/PAH NPG films was measured and shown in Figure 3b. The fluorescence intensity (peak value) of YFP is about nine times stronger on NPG38 than that on glass. After subtracting the fluorescence intensity of acceptor from the total FRET intensity, we estimated the FRET enhancement using formula 2, and the enhancement factor of YFP (Q factor) in the FRET process is shown in Figure 3c. The largest enhancement of the acceptor molecule (YFP) occurs with NPG38, which is 18-fold relative to the YFP fluorescence intensity in FRET on the glass slide. The conversion efficiencies of NPG with different ligament sizes of 27, 32, 38 and 45 nm are 31%, 80%, 90% and 23%, respectively [40,41]. A maximum FRET efficiency of 90% appeared on NPG38, which is consistent with the FRET spectrum in Figure 3a that the maximal acceptor emission intensity and the minimum donor emission intensity appeared on NPG38. Since the spectrum of localized surface plasmon resonance induced by NPG strongly depends on its ligament and pore sizes [32], an optimum ligament size at which the CFP and YFP are best enhanced existed. Additionally, the smaller the ratio between nanopore size to the ligament diameter, the stronger the electromagnetic field due to the coupling effect [37], NPG38 (0.34) has a smaller pore/ligament ratio than the others, and therefore the fluorescence intensity and FRET can be greatly enhanced.

**Figure 4:**
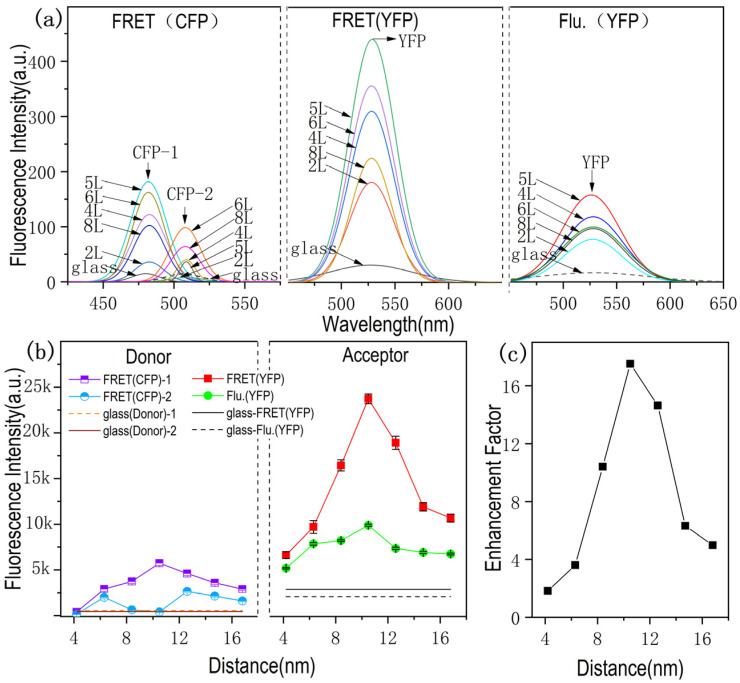
Fluorescence spectra and Enhancement Factor. (**a**) Fluorescence spectra of donor(CFP)-acceptor(YFP) with different distance to NPG 38nm and YFP Fluorescence spectrum of only acceptor at different distance on NPG38. (**b**) The fluorescence intensity of FRET-Donor(left), FRET(Acceptor) and YFP fluorescence intensity (right). (**c**) Enhancement Factor of FRET is calculated by Formula (2).

In order to further study the dependence of FRET enhancement on the distance between fluorescent molecules and plasmonic substrate, NPG38, which shows the highest FRET transfer efficiency, was used for distant dependent FRET measurement. The fluorescence spectra of the donor and acceptor molecular layers were collected on the glass slide and 2L, 3L, 4L, 5L, 6L, 7L and 8L PSS/PAH covered NPG38, and then divided into three characteristic peaks belong to donor (CFP-1 and CFP-2) and accepter (YFP) using Origin peak fitting. The fitted donor (CFP) spectrum and acceptor (YFP) spectrum curves from FRET spectra are shown in Figure 4a together with the fluorescence spectra of YFP only on the substrates.

As shown in the Figure 4a FRET(YFP) and Flu.(YFP), it can be seen that the YFP signal intensity in FRET process is greatly stronger than Flu.(YFP) due to the appearance of donor, and the fluorescence of donor is practically decreased in the presence of acceptor (Figure 4a, FRET-CFP) compared to the fluorescence intensity of CFP only (Figure 2c). With regard to the FRET signal on glass, it can be seen that the FRET process is effectively enhanced by NPG. The fluorescence intensity variation with the distance tuning is shown in Figure 4b, and the intensity of FRET(CFP)-2 obviously decreased when the acceptor exhibited the strongest signal. 

The fluorescence intensity of YFP reached the maximal value with the spacing layer of 10.5 nm, meanwhile the fluorescence intensity of CFP-2 with the wavelength closer to the YFP absorption peak is almost completely quenched (Figure 4b). Therefore, it can be assumed that this longwave emission should be attributed to the sensitized fluorescence of the acceptor molecules, resulting from the energy transfer from the singlet-excited energy donor molecules to the acceptor molecules in the ground state [27].

Figure 4c is the distance-dependent enhancement factor of FRET. The enhancement factor (the ratios between the integrated areas under the spectral region) of YFP from FRET on NPG surface over glass surfaces are plotted as a function of the metal–fluorophore inter-distance (shown in Figure 4c). The maximal enhancement of ~18-fold is observed for the CFP and YFP adsorbed on NPG38 with the probe distance of 10.5 nm from the NPG surface. The enhancement of FRET can be associated both with the direct influence of the plasmon on the efficiency of energy transfer, and with the increase in the fluorescence intensity of the energy donor in the presence of NPG film.

## 4. Conclusions

The distance-dependent plasmon-enhanced FRET in donor–acceptor pairs CFP and YFP on NPG films was studied using LbL-assembled polyelectrolyte multilayers as dielectric spacers. The enhancement is improved upon the introduction of thin spacer layers to prevent non-radiative quenching at small separation distances. We found the maximum increase in the fluorescence intensity of YFP was observed at a distance of 10.5 nm from the NPG with a ligament diameter of 38 nm, revealing that the FRET effect is related to both ligament sizes and spacing distance. We have observed an ~18-fold increase in fluorescence intensity of YFP during FRET at a ~10.5 nm distance relative to that on the glass slide, and a 12-fold and 9-fold fluorescence enhancement of CFP and YFP individually at the same distance of 10.5 nm from the NPG surface. The characteristic energy transfer intensity can be tuned though NPG ligament size and spacing distance, implicating possible applications in nanophotonic devices or sensors. Moreover, the large enhancement of fluorescence emission indicates that FRET based on NPG enhancement has promising applications in light emitting devices and low concentrated analytes detection in chemical and biological systems.

## Figures and Tables

**Table 1:** Component analysis through EDX experiment.

Wt.%	NPG27	NPG32	NPG38	NPG45
Au	97.64	97.52	97.74	98.45
Ag	2.36	2.48	2.26	1.55

Wt.% is the unit of weight percentage.
